# Bringing It All Together: Leveraging Social Movements and the Courts to Advance Substantive Human Rights and Climate Justice

**DOI:** 10.1007/s12142-022-00674-0

**Published:** 2022-12-03

**Authors:** Tracy Smith-Carrier, Kathleen Manion

**Affiliations:** School of Humanitarian Studies, Royal Roads University, 2005 Sooke Road, Victoria, BC V9B 5Y2 Canada

**Keywords:** Climate change, Economic and social rights, Human rights, International environmental law, Justice, Poverty

## Abstract

Although significant literature and jurisprudence has amassed on rights-based climate litigation over recent years, less research and case law has emerged on poverty-related court cases and the fulfilment of economic, social, and cultural rights (ESCR) in Canada. Fewer still are studies exploring the interlinkages between these areas of inquiry. The purpose of this paper is to explore, using Canada as a case study, rights-based developments in climate litigation cases and how these could impact the innovative advancement of ESCR (e.g. to food, housing and water). Typically, issues of justiciability and standing emerge, impeding the realization of such rights. Given the grave threats we now face, climate cases and social movements must be brought together to better hold state actors accountable for their rights obligations. We implore the legal community to explore ways to traverse juridical obstacles to realize the interdependencies of human rights and protect the planet from calamitous climate change.

Adults keep saying: 'We owe it to the young people to given them hope.' but I don’t want your hope. I don’t want you to be hopeful. I want you to panic. I want you to feel the fear I feel every day. and then I want you to act. I want you to act as you would in a crisis. I want you to act as if our house is on fire. Because it is. (Greta Thunberg as cited in Real Leaders 2019)

## Introduction

Anthropogenic climate change poses the single greatest health threat facing humanity today (World Health Organization 2021), one that adds context to other social issues embodied by economic, social and cultural rights (ESCR) infringements (like the rights to food, potable water, and housing). Earlier climate change mitigation efforts largely targeted domestic and international regulatory and policy avenues to legislate greenhouse gas (GHG) emission reductions in countries of both the Global North (Peel and Osofsky 2020) and Global South (Peel and Lin 2019; Rodríguez-Garavito 2020). As these vehicles proved less than favourable, a surge of alternative approaches has emerged, including recent endeavours that have applied legal frameworks, including rights-based approaches, to urge the courts to use their powers to hold governments and private businesses accountable for their emission contributions (Peel and Osofsky 2020; Savaresi and Auz 2019). Given the growing and creative confluence of social movements and litigation calling for climate action, lessons can be learned regarding the legal jurisgenerative approaches emerging that may promote further consideration of state obligations to realize the ESCR of citizens. Therefore, the purpose of this paper is to explore, using Canada as a case study, rights-based developments involving ESCR, and how these shape and intersect with climate litigation cases and the right to a healthy environment. From the perspective of social sciences, using literature on litigation and social movements, we argue that such cases could have reciprocal and positive impacts on the social and legal movements that seek broader social change and rights fulfilment.

We use the case study of Canada, a country with an advanced legal system, as fodder to explore emerging developments in ESCR and climate and environmental law, particularly the right to a healthy environment, and how they might be leveraged to prompt greater action on rights realization. While Canada has seen limited success in this realm to date, it represents a useful case from which to explore the emerging intersections and mutual interests of ESCR with climate litigation, and the broader social movements these could potentially (re)produce. Bringing together resistance strategies, including litigation and other forms of protest, may carry greater weight in calling actors to account for their (in)actions in ensuring the health and sustainability of the future, including both the survivability of all species and the planet (and the quality of this survival). In this article, first, we present the theoretical framework connecting social movements and jurisgenerative approaches for ESCR and climate justice; second, we outline the historical reasoning for the bifurcation of such rights into separate (non/justiciable) domains and the impact this has had on delimiting ESCR and their justiciability in Canada through constitutional, tort and administrative law, and human rights tribunals; third, we consider the rights turn evident in international climate justice cases; and finally, we explore how the pursuit of rights through the courts can impact social movements, historically shown to have the greatest potency as a catalyst for change (Chenoweth 2021). Much of the legal reasoning, logic, and language presented in the juridical corpus of research can be unfamiliar terrain for many social scientists. As social scientists, we seek to bridge this apparent divide, providing the legal, historical and theoretical mooring to tie the role of the courts, political actors and the public in their efforts to reverse the destructive course we now collectively tread.

### Theoretical Framework: Social Movements and Climate Justice

The social movements literature is expansive, illustrating highly diverse strategic decisions that are difficult to definitively or conclusively isolate or explain. Hilson (2002) argues that social movement organizations make the decision to take up a particular strategy (i.e. protest, lobbying or litigation) depending on the political opportunity and legal opportunity present at the given time (e.g. the closedness/openness of the political system and the receptivity of political elites to collective action), as well as structural features (i.e. access to justice through legal funds or legal stock through laws of standing), and access to resources (e.g. financial resources to litigate and legally-trained staff). Previous social movements (e.g. the women’s, environmental, animal welfare and gay and lesbian movements) all employed litigation as a vital strategy to advance their aims (Hilson 2022). Litigation is often adopted because it is perceived to afford a more effective approach for gaining public momentum than other political opportunities (Hilson 2010).

Climate justice and anti-poverty movements are inextricably linked. The realization of ESCR continues to be premised on economic growth, as regressive measures have oft been justified in times of economic contraction (Leishenko and Silva 2014). Should climate change place downward pressure on economies in the future, as it undoubtedly will do, ESCR will be in further jeopardy if they are not firmly ensconced into infrastructure where rights can be claimed and remedies enforced. As the limits of formal equality in redressing entrenched patterns of systemic disadvantage become ever more visible and harmful (Brodsky et al. 2017), an understanding of equality that moves beyond a mere duty of state restraint is needed (Fredman 2005). Arguing for substantive rights fulfilment is key, not solely in the courts, but in the public square. Social movements to date have been singular and disjointed. We argue, as does Matthews (2020), that uniting the collective aims of equity and justice under various causes (e.g. income inequality with environmental and climate) can carry greater weight than the pursuit of any one cause alone.

The strategies used by climate change and social justice social movements have framed narratives and discourses beyond the court (Noonan 2018), attracting media attention, intensifying the pressure on political leaders to act (Gönenç 2019), and presenting vulnerable communities, particularly Indigenous communities, greater avenues for participation and resistance (Benjamin and Seck 2022). Siegel (2004:14) argues that “social movements may well be better vehicles for incubating, articulating, and justifying evolving understandings of the nation’s constitutional values than the regular institutions of democratic governance.” She further states they may also “strengthen law precisely as it unsettles it, enabling and, on occasion, moving [it]” (Siegel 2004: 22). While the views on how effective litigation is as a strategy in the pursuit of social change vary, this process of unsettling holds transformative potential (Gönenç 2019).

### Bifurcated Rights in Canada

Legal decisions on ESCR in Canada have been grounded in an outdated mode of constitutional and human rights reasoning. Built on classical constitutionalism, the country’s bill of rights, the *Canadian Charter of Rights and Freedoms* (hereafter the *‘Charter’*) (1982)*,* has been applied by the courts, almost exclusively, as a negative rights instrument. *Negative rights* are understood to necessitate state protection rather than action (e.g. the right to freedom of speech). Such rights are intended to constrain the state from unduly infringing upon legally circumscribed individual freedoms and liberties (Brodsky and Day 2002). Inclusive of ‘first generation’ civil and political rights ([CPR]; Council of Europe Portal nd), negative rights are differentiated from *positive rights*, as rights requiring state action for their fulfilment (e.g. the rights to food, housing and an adequate standard of living). State actors are required to respect and protect people’s exercise of positive rights, but also take actions to progressively realize their fulfilment. Despite state commitments to meet these obligations, courts in Canada, and more broadly, have been reticent to recognize the justiciability of positive rights.

A breadth of rights commitments were globally affirmed in the *Universal Declaration of Human Rights* ([UDHR] 1948), but a tumultuous and deep ideological and political divide was entrenched in the subsequent core binding international human rights instruments, with negative rights articulated in the *International Covenant on Political and Civil Rights ([ICCPR]*; OHRC 1966b) and positive rights outlined in the *International Covenant on Economic, Social and Cultural Rights* ([ICESCR]; OHRC 1966a). Despite US President Roosevelt declaring ‘freedom from want’ (a positive right) to be one of the four essential freedoms needed to underpin a new global order in the post-World War II era (Alston and Goodman 2013), other US actors led the charge to differentiate these rights because they argued the two sets of rights claims were qualitatively distinct, requiring disparate treatment (i.e. an aspirational texture for ESCR); CPR were necessary immediately to build international law; and although states had the duty to protect rights given the insufficient resources of some, obligations needed to be obtainable (Kirkup and Evans 2009: 226). As such, in contradistinction to the *ICCPR*, weaker language was purposefully employed in the *ICESCR* by the rights architects to appease concerns that it could be applied to encroach on state autonomy by requiring “thicker social programs and a robust welfare state” (Mutua 2016: 136). This cemented the eventuality of lacklustre realization of ESCR (Alston and Goodman 2013). Here, unlike their negative rights counterparts, positive rights were ascribed a subordinate, normative and non-justiciable (Scott and Macklem 1992) status. This bifurcation violates the principles of indivisibility, interdependency and interrelatedness that undergird human rights principles (Whelan 2010), as declared in the *Vienna Declaration and Programme of Action* (OHRC 1993).

Various global actors continue to enliven calls to understand and implement a rights regime that honours the indivisibility of rights. For instance, the UN Committee on Economic, Social and Cultural Rights (1990) affirmed, in General Comment 3, that “the realization of economic, social and cultural rights is an obligation of all States”. Human rights theorists continue to contend that states are obliged to meet ESCR, including Kelley (2008) who suggests that welfare rights are not optional but required obligations of states, and Sen (2009) who argues that ESCR remain worthy goals and ought not to be deemed non-rights. Furthermore, the Maastricht Guidelines on Economic, Social and Cultural Rights (UN 1997) clearly indicate states’ legal requirement to meet their ESCR duties, including “a strong presumption that retrogressive measures on the part of a State are not permitted” (Arif 2019: 201) and that states must meet minimum core obligations.

Despite the pleas of human rights activists, confident in the potential of the ‘indivisible’ and ‘universal’ human rights framework to catalyse the unrealized promises set out in the *UDHR* (UN 1948), subsequent international human rights instruments (and in many state constitutions in countries of the Global North [e.g. Finn 2002] and South [e.g. Pieterse 2004]) have widely been interpreted to be non-justiciable due to the belief that judicial officials do not have the authority to weigh in on political disputes. These matters have been ousted to political actors given the democratic legitimacy afforded them at the ballot box (May 2007). We argue that the increasing intersection of legal and political tenor of social movements offers future hope to realize the indivisibility of the international human rights regime. To date, political actors in Canada, with their extensive legal teams, have spent considerable time and tax dollars persuasively arguing in the courts that legal frameworks that would appear so amenable to granting human rights pose in fact few obligatory duties on them (Brodsky and Day 2002). In the disavowal of such rights, however, structural problems (e.g. poverty, homelessness, hunger and climate insecurity) have proliferated (Smith-Carrier et al. 2020; Smith-Carrier 2021), and rights-holders experiencing the most severe systemic discrimination and impoverishment have been denied access to justice (e.g. Smith-Carrier et al. 2017). Consequently, Canada, although positioning itself as a global beacon of human rights (Ignatieff 2000; McLeod 2017), has maintained a poor domestic record on human rights (de Schutter 2012).

Canada’s less than sanguine approach to ESCR was typified in the quashing of a right to an adequate standard of living, as ruled in the *Gosselin v. Québec (Attorney General)* (2002) case. *Gosselin* set the stage for how ESCR in Canada would come to be interpreted (Jackman 2019). In 2002, the Supreme Court of Canada dismissed Louise Gosselin’s *Charter* challenge related to a welfare regulation in the Province of Quebec that limited benefits for those under 30 years of age to two-thirds of the existing welfare benefit (a paltry $170/month relative to the $466/month regular benefit), claiming the regulation was age-discriminatory and violated the (*Quebec* and) *Canadian Charter*, under Sect. 7, guaranteeing life and security of the person, and Sect. 15, the equality provision (Brodsky 2003).

The Supreme Court, in upholding the lower and appeal court rulings, rejected the notion that Sect. 7 of the Canadian *Charter* imposed positive rights duties on governments, and deemed that the reduced benefit was not in violation of Sect. 15 (prohibiting age discrimination), given that the differential benefit structure was ameliorative, and intended to enhance the position of young people in Quebec by helping them find employment, allowing them to lead, ostensibly, richer and more independent lives. The court deemed there to be insufficient evidence to support the claim under Sect. 7, arguing that this provision affirmed a negative guarantee restricting the state from depriving people of life, liberty or security of the person (Murdoch 2002).

Regrettably, the *Gosselin* case took Canadian constitutional law (“two steps”) backwards (Jackman 2019). It placed a more onerous evidentiary burden on people living in poverty than on the well-financed government defendants (Smith-Carrier et al. 2020) and failed to discredit the “uncritical and stereotype-infused approach” (Jackman 2019: 103) to the evidence presented. Justice Paul Reeves of the Quebec Superior Court articulated his reasoning on *Gosselin* this way (translated from French):

Studies show that the majority of the poor are poor for intrinsic reasons. They are under-educated or psychologically vulnerable people, or people who have a weak work ethic (Jackman 2019: footnote 126).

Justice Reeves, and later former Chief Justice Beverley McLaughlin of the Supreme Court, anchored the ruling to an individual explanation of poverty, which sees financial hardship as arising from personal deficits (e.g. laziness, a lack of motivation, mental health disorders, substance use issues), rather than from systemic determinants (e.g., a sluggish economy, global pandemics, a dearth of quality jobs, housing or childcare). Yet a significant evidentiary base has now accrued pointing to the structural origins of poverty (Smith-Carrier et al. 2021), particularly from the burgeoning social determinants of health literature (Raphael 2006). *Gosselin* laid the framework, however, for how all future cases related to poverty (and other positive rights) in Canada would thereafter be interpreted (Jackman 2019).

Despite the ruling of non-justiciability in *Tanudjaja v Attorney General* (2014) (the ‘right to housing’ case in Canada; Heffernan et al. 2015), recent Canadian legislation has, for the first time, explicitly affirmed the right to housing through the passage of the National Housing Strategy Act (2019). The act explicitly takes a rights-based approach, consonant with the *ICESCR* (OHRC 1966a). Despite this landmark victory for rights advocates, the federal government has dedicated relatively little funding to housing in the years leading up to and since the act’s passage. Expenditures on affordable housing in 2018 represented only 0.8% of the overall federal budget, one of the lowest years on record since the 1970s (Gold 2019). Although federal housing funding has increased since 2019, it remains grossly inadequate to meet the intensifying demand, particularly in the wake of the COVID-19 pandemic (Parliamentary Budget Officer 2021) and crippling lack of affordable housing across the country. Indeed, Canada’s non-market housing sector (reserved for those living with low incomes) equates to a mere 4% of its overall housing stock, compared to 10–30% in many other countries of the North (Hulchanski 2021). The newly appointed Federal Housing Advocate, working alongside the National Housing Council and attendant review panels, will therefore have significant work to do to urge the state to actively fulfil the right to housing, using the maximum resources available (Biss et al. 2022).

Given the impasse on the justiciability of ESCR, some legal scholars have increasingly called on human rights tribunals to order effective remedies to redress entrenched patterns of discrimination and inequality in Canada (Brodsky et al. 2017). The trajectory of the 2007 Assembly of First Nations and *Canada (Attorney General) v. First Nations Child and Family Caring Society of Canada *(Jacklin and Eidse-Rempel 2021) case is instructive in this regard. In 2016, the Canadian Human Rights Tribunal issued a series of remedial orders to the federal government pursuant to its decision that the state had indeed discriminated against First Nations on-reserve and in the Yukon by chronically underfunding child welfare services. Rather than responding forthwith, the government dragged its heels, refusing to comply. The case in effect demonstrated that although administrative tribunals (i.e. the Canadian Human Rights Tribunal) have broad scope to order systemic remedies, the state’s unwillingness to defer to their authority renders the enforcement of such orders difficult (Cave 2021). Looking beyond Canadian borders, this case occurred in the aftermath of *Demanda Generaciones* in Colombia, where the success of the case was largely eclipsed by the Colombian government’s failure to execute the court’s orders expeditiously and effectively (Parker et al. 2021).

After a lengthy wait, in response to *Canada (Attorney General) v. First Nations Child and Family Caring Society of Canada* (Jacklin and Eidse-Rempel 2021)*,* the Government of Canada released an Agreement in Principle to set aside $40 billion for compensation and reform of the First Nations child welfare system, setting a record in such agreements and opening an avenue for outlining the fiduciary responsibility of the state to ensure ESCR of Indigenous children in Canada without discrimination (Indigenous Services Canada 2022). In addition to this case, some success has also been garnered in arguing for the right to safe drinking water, a right intrinsic to but unstated in Article 11(1) of the *ICESCR* (OHRC 1966a). In July 2021, the Government of Canada settled *Tataskweyak Cree Nation *et al*. v. Canada (Attorney General)(*2021*) * on the basis of inaction in response to ongoing and prolonged drinking water advisories in multiple First Nations communities across Canada. The class action resulted in, among other actions, compensation of $8 billion being rendered for individuals deprived of clean drinking water and a commitment of $6 billion to support reliable water access on reserve moving forward (McCarthy Tetrault 2021).

### The Rights Turn in Climate Litigation Abroad

The increasing frequency of climate-related disasters will result in significant migration, displacement, food systems disruptions, homelessness and extreme poverty (IPCC 2022). Particularly evident in climate justice cases is *“*a ‘turn to rights’, both in the sword and shield cases” (Krommendijk 2021: 15). Setzer and Byrnes (2020) identified no less than 1587 cases of climate litigation that were brought forward internationally between 1986 and May 2020, including 1213 cases in the USA alone. Thirty-four cases have been launched in Canada since 2018 (Climate Change Litigation Cases 2022a). Climate jurisprudence is thus rapidly evolving and expanding, making the courts an increasingly viable tool to complement other avenues to demand climate justice (Peel and Lin 2019). Legal decisions related to similarly situated ECSR could benefit from the ‘rights turn’ apparent in litigative climate cases underway around the globe (Peel and Osofsky 2018; Setzer and Vanhala 2019).

Given repeated affirmations that climate change adaptation and mitigation proposals fall under the purview of political actors, not the courts, a significant lacuna has been created in the juridical enforcement and accountability of environmental regulations internationally. Like ESCR, until recently, climate and environmental rights have been presumed to be policy matters best left in the hands of political actors (May and Daly 2020). Recent litigation cases around the globe, however, are testing the bounds of justiciability, and although international fora have not yielded constructive spaces for climate litigation, the courts are increasingly being seen as a viable venue through which state and non-state actors can be held to account for their contributions to harmful climate change (Beauregard et al. 2021). Although international environmental law has a different corpus than human rights law, there are increasing examples of case law using claims to environmental and human rights concomitantly, particularly for children, to argue a person’s right to a healthy environment, for instance in Uganda, Nepal, Colombia, Pakistan and Norway (Climate Change Litigation Databases 2022b). This has opened the door to nascent but growing jurisprudence within different global domestic courts to the justiciability of positive rights, including the indivisibility of rights-based frameworks arguing for state responsibility to ensure the right to a clean and healthy environment; a right recently endorsed by Member States at the UN General Assembly (UN News 2022).

Rights-based claims employed in climate litigation cases internationally have tended to place particular emphasis on *inter-generational equity*, the notion that owing to the (in)actions of the current and preceding generations, future cohorts of children and young people will be burdened with the most adverse consequences of climate and environmental destruction (Davies 2020) (as first successfully argued in *Minors Oposa v Factoran* in the Philippines in 1992; UN Environment Programme 1993); *intra-generational equity*, recognizing that the worst impacts of climate change will be borne by those who have contributed the least to it and have the least adaptive capacity to withstand its catastrophic effects (Lewis 2021); or on the indivisibility of positive and negative rights.

Being potentially younger than the established voting age and therefore denied a voice in existing democratic processes, some children and youth (including many from Indigenous communities) have initiated climate litigation as a vehicle for agency, allowing them to voice their dissent to actions that maintain the status quo (Daly 2022; Parker et al. 2021). Parker et al. (2021) observe three lines of reasoning in these youth-based climate-related cases: insufficient efforts to reduce carbon emissions and meet climate commitments (e.g. *ENJEU* in Canada*, Juliana v. United States*) (2020); insufficient efforts to implement mitigation and adaptation policies (e.g. Climate Change Litigation Databases (2018); and judicial review of regulatory approaches (e.g., *PUSH* Sweden). Instructively, recent cases (e.g. *La Rose v. Canada; Juliana v. United States*) have also focused on failures of states related to the public trust doctrine (that claims that states hold natural resources, like navigable waters, in trust for the good of their citizens). The efficacy of this doctrine was asserted in the US by Justice Walters in *Chernaik v. Brown* (2020) stating, “This court can and should issue a declaration that the state has an affirmative fiduciary duty to act reasonably to prevent substantial impairment of public trust resources.” However, in international courts, results have been equivocal. The judge in *La Rose*, for example, ruled that the public trust doctrine is not enshrined in Canadian law and that climate change is ‘too political’. Upon appeal (awaiting oral hearing at the time of writing), the appellants argued that public trust is enshrined in common law and that “justiciability is not about assessing how far-reaching the political or societal ramifications of the resolution of a claim may be” (*La Rose v. Canada* 2021).

Successes have been recorded in *Demanda Generaciones*, as the court sided with the young plaintiffs who argued that the Colombian government be held accountable for its failure to reduce deforestation in the Amazon, congruent with its zero-net target for 2020 (Parker et al. 2021). Orangias (2021: 580) highlights the important contribution that the Supreme Court in Colombia made in this case*,* stating “natural resources are shared by all inhabitants of Planet Earth, and by their descendants or future generations who do not yet have a physical hold of them.” Moreover, in *Neubauer et al. v. Germany* (2021) the court struck down the German Federal Climate Change Act for its violation of the plaintiffs’ constitutional rights, claiming that it maintained emissions reductions deemed to unfairly burden future generations (Parker et al. 2021). A landmark victory was recorded too in *Leghari v. Federation of Pakistan*, after the court held the government of Pakistan responsible for its failure to address climate change by delaying the implementation of the country’s climate policy framework (Peel and Osofsky 2018).

Litigation cases seek to hold not only state actors accountable for their emission contributions, but non-state actors also. In *Urgenda Foundation v State of Netherlands *(2015), the court decided that the Dutch government had violated its duty to care for its citizens by failing to take appropriate actions to protect them from the effects of climate change (Krommendijk 2021); a ruling subsequently brought to the Dutch Court of Appeal, and upheld, in 2019, by the Dutch Supreme Court (Peel and Osofsky 2020). The court expanded upon the *Urgenda* decision on April 5, 2019, in *Milieudefensie (Friends of the Earth Netherlands) v. Royal Dutch Shell* (“Shell”) (Climate Change Litigation Databases 2019) by holding the private corporation accountable for its contributions to climate change, in violation of the Dutch Civil Code, and international human rights treaties (Climate Change Litigation Databases 2019; Peel and Osofsky 2020). The Hague District Court ruled, on May 26, 2021, that, under the unwritten standard of care laid out in the Dutch Civil Code (affirming that acting in conflict with what is generally accepted according to unwritten law is unlawful), Shell had violated its obligations to prevent dangerous climate change, ordering the corporation to reduce its emissions by a net 45% by 2030, relative to 2019. It went a step further and made its decision ‘provisionally enforceable’, meaning that even if the case is appealed, Shell would still be required to meet its reduction obligations (Climate Change Litigation Databases 2019).

In applying the indivisibility argument, the Inter-American Court of Human Rights has begun to recognize environmental rights as independently justiciable from CPR, with *Lhaka Honhat v Argentina* (2020) paving the way for the justiciable right to a healthy environment, as well as more general rights to ESCR as secured in Art. 26 of the American Convention on Human Rights (also affirmed in *Lagos del Campo v Peru) (*2017*)* (Mejía-Lemos 2022). While the judges were split, Judge Pazmiño Freire stated that the court had overcome a “’restrictive narrative’, which established a ‘discriminatory hierarchy’ among human rights, unduly excluding economic, social, cultural and environmental rights from enforcement” (as cited in Mejía-Lemos 2022: 321). This was a landmark case for linking environmental and ESCR by drawing on “the interdependence of human rights; (and) vulnerability as a factor compounding the implications of environmental damage” (Mejía-Lemos 2022: 323). In this finding, the court held that the progression of positive rights could be enforced with their negative rights obligations, as the environmental damage of forestry interfered with the “effective enjoyment of all human rights” (Mejía-Lemos 2022: 323).

In September 2022, the UN Human Rights Committee (2022) broke new ground, opening the door to environmental rights protection through the human rights framework for Indigenous people living in low lying areas and offering some approaches for securing positive rights obligations. It found that the Australian government had failed its obligation to protect the cultural rights of Torre Strait Islanders under the *ICCPR* (Art. 6, 17, 24 (1) and 27). The Committee found that the state had been responsible for GHG emissions, had failed to mitigate their impact and failed to meet obligations by not upgrading seawalls thereby putting vulnerable communities at risk for climate-related disasters. The plaintiffs argued that this impacted their traditional way of life (*Daniel Billy and others v Australia* [Torres Strait Islanders Petition] 2019). While it struck down claims on the basis of the right to life with dignity (Art. 6), it found relevance to claim that the population was vulnerable to extreme weather events and could not reasonably be able to afford measures to mitigate impacts on its own. The Committee stated,

While the State party notes that socioeconomic entitlements are protected under a separate Covenant, the Committee observes that the preamble of the present Covenant recognizes that the ideal of free human beings enjoying freedom from fear and want can only be achieved if conditions are created whereby everyone may enjoy their civil and political rights, as well as their economic, social and cultural rights (HRC 2022: 13).

The Committee went on to say that “by failing to discharge its positive obligation to implement adequate adaptation measures to protect the authors’ home, private life and family, the State party violated the authors’ rights under article 17 of the Covenant” (HRC 2022: 13).

These recent cases illustrate the preconceived notion that rights ascribed to the *ICESCR* are inherently non-justiciable (because they are political not legal; Erratum to 2021) is increasingly being challenged. As Cameron and Weyman (2021: 200) argue, the lack of clarity in what is political and what is legal “goes a long way to explaining the lack of clarity and coherence in Canada’s political questions doctrine.” The absence of progress may prove useful as the tack to argue for greater action. Previous approaches that have had limited success, particularly within the realm of environmental rights, have linked the failure to progressively provide positive rights with non-compliance to secure CPR even as it relates to ESCR. Legal arguments then focus on who is the duty bearer of progressive implementation and the focus of legal decision-making as the duty bearer (Arif 2019).

In observing three fruitful cases abroad (i.e. Australia’s *Sharma by her litigation representative Sister Marie Brigid Arthur v. Ministry for the Environment* (2021), *Neubauer*, *Shell*), Peel and Markey-Towler (2022: 1485) outline the ingredients for strategic climate litigation success: (a) thoughtfully selecting plaintiffs to communicate a strategic message; (b) drawing upon an experienced legal team with a track record for advancing strategic climate legal interventions; (c) targeting defendants widely considered to be laggards in their climate action; (d) grounding legal arguments on the most current climate science; (e) employing innovative legal arguments, including those highlighting duties of protection; and (f) calling for remedies that extend beyond the circumstances of the individual litigants and contribute to greater policy and regulatory changes.

Canada’s political system as a federation “complicates the ability to comprehensively tackle climate change, and has led to historical and current lags in Canada’s response to climate change” (Choquette et al. 2021: 154). International treaty commitments on climate change have, to date, been equivocal (e.g. Smith-Carrier and On In Press). In 2002, for instance, the federal government committed to the provisions set out in the Kyoto Protocol, but in an about-face, later notified the *UN Framework Convention on Climate Change* that it intended to exercise its right to withdraw from it. Then, in 2016, the federal government committed, as signatory to the Paris Agreement, to reduce its GHG emissions by 30% below 2005 levels by 2030 (Environment and Climate Change Canada 2021); a pledge it later increased to 40–45% (Government of Canada 2021). However, this commitment may be beyond the country’s grasp (Chung 2021), given the business-as-usual approach that appears to have been ensconced (Smith-Carrier and On In Press).

Although some cities (e.g. Victoria and Vancouver; Jost et al. 2020) and provinces (e.g. British Columbia; Dale et al. 2020) are showing greater progress than others, overall climate adaptation and mitigation climate actions in Canada have been lacklustre. In fact, Environment and Climate Change Canada (2019) has revealed that Canada is warming at twice the rate of the rest of the world. Given growing recognition of the urgency of climate action, in 2019, the Canadian House of Commons declared a national climate emergency (Parliament of Canada 2019); a pronouncement that has since reverberated across 517 municipalities across the country (iPolitics 2021). This has inspired, as previously noted, a range of climate activists, legal scholars and youth to explore various approaches of redress, including prompting a juridical response.

Climate litigation cases in Canada have, to varied degrees of success, applied both constitutional and human rights provisions to ground their claims. These largely youth-led, rights-based cases may prove critical in breaching the seemingly impenetrable impasse of justiciability (Cameron and Weyman 2021). Regrettably, the court rendered a determination of non-justiciability in *Friends of the Earth v. The Governor in Council and Others *(2009), a case asserting that the Canadian government had failed to comply with the Kyoto Protocol.

Notwithstanding the decision reached in *Friends of the Earth *(2009), more recent climate cases in Canada have not been thrown out on non-justiciable grounds. Young people, in *Mathur v. Ontario *(2021), took the provincial government to court for legislating an end to the province’s cap and trade program, given what they perceived to be a violation of their constitutional rights under *Charter* Sects. 7 and 15. As is customary, the government defendants argued that the matter was non-justiciable, claiming that climate change is “notoriously planetary in scope”, too complex to be measured (Cameron and Weyman 2021: 7), and that the plaintiffs offered unsubstantiated speculations about the consequences of the government target for the future. The Ontario Superior Court of Justice however rejected Ontario’s motion to strike out the young peoples’ claims on the basis they were non-justiciable, and the case will proceed to a full hearing (Cameron and Weyman 2021).

Court cases involving Indigenous-led resistance to fossil fuel projects that expressly aim to assert Indigenous self-determination could also be seen as human rights-based climate litigation (e.g. opposition to the Trans Mountain pipeline through *Tseil-Waututh Nation v Attorney General of Canada* (2018)) in seeking to call Canada to account for its procedural duty to consult and accommodate Indigenous peoples under constitutional law. The ruling in *Tseil-Waututh*, under appeal, concluded that this duty was met, leaving Indigenous climate activists little recourse but to continue in the courts (oft a financially costly option) or engage in protest (a tactic that leaves them open to an injunction or arrest). The duty to consult and accommodate, however, could be invoked in future projects that involve policies that engender GHG emission increases (e.g. pipeline expansion projects) (Benjamin and Seck 2022).

Another avenue for litigants to consider when the promise of rights realization through administrative law appears to ring hollow is through tort law. In *Nevsun Resources Ltd v Araya *(2020), specifically querying whether a private, non-state actor can be held liable in Canada for alleged breaches of international law abroad, the appellate court ruled that indeed it could. In the court’s decision on *Araya*, Justice Abella affirmed that since.

(I)nternational law not only percolates down from the international to the domestic sphere, it...also bubbles up, there is no reason for Canadian courts to be shy about implementing and advancing international law... (Gowling 2020: para. 41).

The *Nevsun* case involved crimes against humanity and the prohibition of slavery, alleged acts deemed of such fundamental importance that they ought to be characterized as *jus cogens* (entrenched norms from which there can be no derogation; Muchlinski 2020). Non-justiciability here was not evoked, as “the prohibition against cruel, inhuman and degrading treatment was an absolute right which no social goal or emergency could limit” (*Nevsun Resources Ltd. v. Araya* 2020*:* para 103). Whether the case is settled in or out of court (cases involving multinational corporations often engage the latter), “the Supreme Court will have paved the way towards making Canadian corporations warier of human rights litigation risk in the context of their overseas operations” (Muchlinski 2020: 527).

### Justiciability and Standing

Climate litigation and rights-based cases in Canada, as elsewhere, have frequently been dismissed owing to admissibility concerns, including a lack of standing (the determination that an individual/group does not have the right to bring an action before the court) and non-justiciability. The *ENJEU* class action, for example, was dismissed because the age definition of the class of residents (under age 35) was deemed to be arbitrary and not objective. Given concerns that the class action would place a burden on parents to make litigation decisions for their children, and that Environnement Jeunesse (2019) was not an appropriate plaintiff, Justice Morrison determined that the class action was not the appropriate procedural channel for the case (Feasby et al. 2020). Similarly, out of 21 youth-led, rights-based cases brought forward in countries around the world, 18 of them were dismissed at the preliminary stages (some, however, are now under appeal) due to a finding of non-justiciability or a lack of standing (Parker et al. 2021).

In contrast, in the poverty-related *Gosselin* case, although not dismissed at the preliminary stage owing to a lack of standing, Justice Reeves took issue with the fact that Louise Gosselin was the sole witness representing the entire class of recipients affected by the social assistance regulation and accepted the government’s depiction of the evidence and expert reports about the circumstances of other young welfare recipients as hearsay. He also criticized the lack of evidence presented on the comparative group (over age 30) receiving the full benefit amount (Jackman 2019: 91). In Canada, interpretations of the *Charter* as being a negative rights instrument cast a long shadow, over three decades after *Gosselin*, virtually every poverty-related case (e.g. homelessness; the *Tanudjaja* case) applying a positive rights claim has been dismissed on non-justiciable grounds, leaving people living in poverty no assurance that the *Charter* offers them any meaningful rights to ‘life, liberty and security of the person’ (Jackman 2019).

Justiciability, a deeply contested concept, has received ample attention in the literature (e.g. Langford 2008). Given that, as the Supreme Court of Canada notes, “there is no single set of rules” surrounding justiciability, its scope remains poorly delimited and subject to significant legal discretion (Chalifour et al. 2021: 37). In theory, perceptions of over-reach are well-founded, as the courts ought not exceed their bounds and intervene in policy matters most appropriately determined by duly elected political actors. In practice, however, concerns of justiciability have led to a deference to state authority that defies any trace of accountability (Fredman 2006). To bring forward rights-based claims, applicants must first demonstrate that they have standing as ‘victims’ who have suffered an injury(ies) because of an act or omission of the state (Lewis 2021). Cases dismissed due to a lack of standing can deny access to justice to those most vulnerable to the adverse effects of neoliberal processes (i.e. systemically impoverished people, children/youth and Indigenous peoples). Deprived not only of the substantive remedies that could redress the ESCR (and climate and environmental rights) violations they have suffered, they are denied the right to even be heard. This erasure of agency, and the silencing that ensues from it, is also problematic as it shields recalcitrant states from justifying their (in)actions as principal duty-bearers of human rights (Parker et al. 2021).

The framing of Charter-based claims appears to be important. In *La Rose v Canada *(2019), the plaintiffs raised objections to the government’s conduct and inadequate GHG reduction targets writ large, whereas those in *Mathur v Ontario* (2021) tied inadequate GHG targets to specific Ontario legislation, drawing attention to the regulatory inadequacies of provincial legislation that stymie progress on meeting national and international commitments. Benjamin and Seck (2022) thus argue that narrower claims may have greater chances of success than those situated on broader claims that have erstwhile invited a non-justiciability ruling. In considering *Juliana*, the authors highlight how the plaintiffs, in amending their claim, have requested a declaratory judgement by the court that national climate action is insufficient. This tactic is one that plaintiffs in Canada could consider in asking the courts to take a more progressive interpretation of the *Charter* through limiting their request for effective remedy. Such a judgement might then allow the courts to leave it to political actors to determine how the state would meet its obligations (Benjamin and Seck 2022).

### Bringing It All Together: Social Movements and the Courts

Advancing an agenda of permanent austerity and welfare retrenchment (Pierson 2002), neoliberal states, including Canada, have embraced a callous disregard for ESCR, and eschewed their redistributive role (Riches 2002) using specious claims of strained budgets and insufficient resources (Elson 2012). The deferral by the courts to the executive branch has thus accommodated the prevailing neoliberal orthodoxy at the expense of rights (Cohen and Dagenais 2021) and sanctioned ameliorative programming that by design is under-inclusive and discriminatory (Gary et al. 2010). As demonstrated above, political actors in Canada have not taken their role as duty-bearers of rights seriously, and there is little recourse to force their hand (Smith-Carrier et al. 2020). Human rights champions that look to the rights’ edifice as the best chance of overhauling a myriad of systemic issues must therefore grapple with the inherent and yet unresolved gaps and tensions in the legalistic-rights framework (Mutua 2016). The rights-turn in the handful of climate cases described herein (e.g. *Urgenda*, *Friends of the Earth* and *Shell)*, including in Canada (e.g. *Mathur*), provide modest room for optimism as they appear to have transcended the boundaries of non-justiciability that have beleaguered positive rights to date.

To dismantle and reverse systemic penalties, and to better include and invite members of systemically impoverished groups to participate on equal footing with others, positive provision is required (Lister 2013). Indeed, CPR (e.g. the right to run for office) are essentially meaningless to the hungry and homeless struggling to survive. The notion that elected governments only, and not judiciaries, have the “absolute and exclusive legitimacy to decide on questions of resource allocation is a sham” (Desai 2009: 25). Insisting that positive actions against want and need are the province of substantive human rights, as Fredman (2005) succinctly argues, requires that their avowed quintessential political character be revisited. Rather than interfering in political processes, the prerogative of the courts to demand state actors provide reasoned justification for their distributive and climate-related decisions would in fact anchor and enhance the accountabilities of democratic governance. The way forward then is not judicial deference, but judicial intervention in a way that would ensure state and non-state actors are answerable for their (in)actions and required to rigorously defend them (Fredman 2005). In fact, the more that individuals lack agency in an ostensive democratic society, the more judicial intervention is needed (Desai 2009).

Judicial leadership is necessary to recognize the urgency of the moment and offer legal protections that will compel political actors to act to protect their citizenry (Carlarne 2021). This leadership void is immense and requires that judiciaries take seriously their role in ensuring justice. Climate destruction and the social inequalities wrought by it, will invariably threaten the principles of democracy, rule of law and fundamental rights of people that undergird advanced legal systems. Fortunately, climate litigants are learning, using the variety of legal channels at their disposal, how to present the complexity of climate evidence to the courts, including the causal connections to make, the remedies to claim and the strategies that will be persuasive moving forward. As the evolving litigation matures, it is imperative that the judiciary embrace its role as a co-equal branch of government and recognize that it is “judicial intervention that will realign state responsibilities with fundamental rights” (Carlarne 2021: 11).

Climate litigation cases have influence beyond their immediate jurisdictional boundaries, and as Bouwer (2018) reminds us, the relative size of the court or the case, however banal it might appear, may hold instrumental sway. As foreign precedents can and do influence domestic legal decisions (Gentili 2013), advancements in rights-based cases internationally could generate ripple effects in Canada. Earlier research on legal mobilization suggested that litigation was not a particularly helpful strategy in generating the kinds of political changes litigants sought to engender. This legacy has led to a veritable dearth of attention to the impacts of litigation on social movements to date. Yet such cases can have a spillover, indirect, or what Galanter (1983) refers to as radiating effects, which can influence social movements. They do so by offering legal argumentation that can be used by and for courts elsewhere, including in comparative legal analyses; by raising the expectations of movement constituents and encouraging them to press on in their collective change efforts; and by prompting press coverage, increasing the public’s awareness about the issue(s) and their ability to exert pressure on political actors to respond (Boutcher and McCammon 2019).

Climate litigation may or may not be effective in generating the radical restructuring necessary to subvert, restrain or tame the global capitalist system (Stuart et al. 2020; Wright 2010) that is bringing humanity ever closer to the brink of the disastrous tipping point the World Meteorological Organization ([WMO] 2022) reckons we may now have reached. Indeed, while the legal community continues to debate whether (positive) rights have any utility beyond their formal exercise, it is clear “we are heading in the wrong direction” (WMO 2022). Climate and other rights-based litigation is but one lever to prompt change, yet as some (e.g. Marjanac 2020) have persuasively argued, such cases may be more instrumental in drawing greater attention to the necessity of change than could be garnered from their legal outcomes, favourable or not. They can shape the moral arguments that have been shown to have greater weight than economic or social proposals in prompting the necessary public outcry to build and sustain social movements (Barry et al. 2013; Han and Ahn 2020). Their valency is also impacted when cross-pollination and convergence occur across social movements, uniting advocates in the struggle for seemingly disparate, but deeply intertwined goals (e.g. ending poverty, gender inequality and racial justice) to confront the inequitable, unjust and environmentally destructive forces of the global capitalist political economy (Tramel 2018).

Nicholson and Chong (2011) describe human rights bandwagoning as a means of buoying existing climate and social justice activism. The bandwagon, driving change efforts, enables critical connections to be made between power asymmetries and injustice that are at the centre of climate destruction. Those set on harnessing its benefits recognize, as do Nicholson and Chong (2011), that action on climate change is not possible without also addressing basic economic, social and political inequities. Climate change is at once cause and consequence of the non-fulfilment of rights; it is because the rights of people and planet have been so repeatedly and egregiously violated that harmful environmental practices have been legitimated to ensue. Civil, political, economic and social rights will have no meaningful exercise in the absence of the right to a safe environment. The disavowal of such rights will lead to growing political instability and civil unrest, threatening protected CPR under the *ICCPR*, and to growing market failures, hunger and deprivation, compromising ESCR. ESCR are indivisible from CPR and must be afforded equal substantive treatment for both to be reinforced and sustained (Nicholson and Chong 2011).

The normative and rhetorical tools provided by human rights offer shared language, tropes, and (re)framing devices that help initiate, modify, and amplify arguments for change. The *Urgenda* decision, for example, offered a powerful success in climate litigation, but it also appears to “have shifted the debate over climate politics in the Netherlands” (Jodoin et al. 2018: 175). Litigation is therefore an essential instrument to foment social movements, used for its political and social power as much as its potential for legal enforcement. With the global upsurge in the present ‘social movement society’ (Jenkins et al. 2014), work must be done to centre the common roots of, and ‘convergent space’ within (Routledge 2003), social formations, allowing for better marshalling of resources and a broadening mobilization (Smith 2012). As Harvey (1996) has argued, social movements must develop a universalist politics that transcends particularist exigencies to find a common ground for transnational solidarity.

## Conclusion

The Supreme Court acknowledged that the *Charter* should be regarded as a “living tree” …with “the possibility of growth and adjustment over time” (*B.C. Motor Vehicle Act* 1985). Similarly, former Chief Justice McLachlin, in *Gosselin,* conceded that “one day s. 7 (of the *Charter*) may be interpreted to include positive obligations” (Jackman 2019: 93), opening the door for future claims to apply Sect. 7 to advance ESCR. The day has come to call for new international norms that embrace the indivisibility of CPR with their ESCR counterparts, and that recognize the intersections in and between this assemblages of rights with the right to a healthy environment. Concomitantly, the legal argumentation applied in the courts, with their radiating effects across borders, may provide the (re)framing devices that might enable a more united and expansive justice movement to take shape.

Successes have so far been scant within the Canadian context, the growing range of cases and legal reasoning employed is illustrating both the growing creativity of legal advocates, but also the public energy to use litigation as a tool for policy change. As a case study, it is also useful to look beyond our borders to an increasingly conversant international human rights community. A juridical response is of the many needed approaches to redress the existential crisis imposed by the nexus of poverty, inequality and the climate crisis, albeit it offers a veritable path forward. The impacts of climate litigation are not limited to the direct effects they engender in changing legal frameworks but include the indirect effects that have been shown to catalyse change processes. As the fate of humanity and the planet hangs in the balance, we implore the legal community to recognize their role as co-equals to government to realize the interdependencies of human rights and protect the planet from calamitous climate change.

## References

[CR1] Alston P, Goodman R (2013) International Human Rights. Oxford University Press, Oxford

[CR2] Arif SMA (2019) Economic, social and cultural rights of women. International Journal of Law and Management 61(1):191-204. 10.1108/IJLMA-01-2018-0002. Accessed 3 Feb 2022

[CR3] *B.C. Motor Vehicle Act* (1985) 2 S.C.R. 486

[CR4] Barry J, Mol AP, Zito AR (2013) Climate change ethics, rights, and policies: An introduction. Environmental Politics 22(3):361-376. 10.1080/09644016.2013.788861. Accessed 20 Mar 2022

[CR5] Beauregard C, Carlson DA, Robinson SA, Cobb C, Patton M (2021) Climate justice and rights-based litigation in a post-Paris world. Climate Policy 21(5):652-665. 10.1080/14693062.2020.1867047. Accessed 13 Feb 2022

[CR6] Benjamin L, Seck SL (2022) Mapping human rights-based climate litigation in Canada. Journal of Human Rights and the Environment 13(1):178-211. 10.4337/jhre.2022.01.08. Accessed 27 Oct 2022

[CR7] Biss M, Porter B, Raza, S, Desbaillets D (2022) Progressive realization of the right to adequate housing: A literature review. The National Right to Housing Network. https://housingrights.ca/wp-content/uploads/NHC-Progressive-Realization-Paper_EN.pdf. Accessed 20 Nov 2022

[CR8] Boutcher SA, McCammon HJ (2019) Social movements and litigation. In: Snow DA, Soule SA, Kriesi H, McCammon HJ (eds) The Wiley Blackwell companion to social movements (2nd ed.) Wiley-Blackwell, New Jersey, pp. 306-321

[CR9] Bouwer K (2018) The unsexy future of climate change litigation. Journal of Environmental Law 30(3):483-506. 10.1093/jel/eqy017. Accessed 25 Feb 2022

[CR10] Brodsky G (2003) Gosselin v. Quebec (Attorney General): Autonomy with a vengeance. Canadian Journal of Women & the Law 15(1): 194. https://povertyandhumanrights.org/wp-content/uploads/2021/03/paper_jan_04_v51.pdf. Accessed 15 Feb 2022

[CR11] Brodsky G, Day S (2002) Beyond the social and economic rights debate: Substantive equality speaks to poverty. Canadian Journal of Women & the Law 14:185. https://povertyandhumanrights.org/docs/11_DAY_BRODSKY.pdf. Accessed 16 Feb 2022

[CR12] Brodsky G, Day S, Kelly F (2017) The authority of human rights tribunals to grant systemic remedies. Canadian Journal of Human Rights 6:1-48. https://cjhr.ca/the-authority-of-human-rights-tribunals-to-grant-systemic-remedies/. Accessed 16 Feb 2022

[CR13] Cameron C, Weyman R (2021) Recent youth-led and rights-based climate change litigation in Canada: Reconciling justiciability, Charter claims and procedural choices. Journal of Environmental Law. 10.1093/jel/eqab026. Accessed 17 Oct 2022

[CR14] *Canadian Charter of Rights and Freedoms, *s 7, Part 1 of the* Constitution Act, 1982, *being Schedule B to the Canada Act 1982 (UK), 1982, c 11

[CR15] Carlarne CP (2021) The essential role of climate litigation and the courts in averting climate crisis. SSRN. https://papers.ssrn.com/sol3/papers.cfm?abstract_id=3761850. Accessed 18 Oct 2022

[CR16] Cave J (2021) Remedies matter: Evaluating the efficacy of remedies in public law litigation for executive action. Dalhousie Journal of Legal Studies 30:1-32. https://digitalcommons.schulichlaw.dal.ca/cgi/viewcontent.cgi?article=1423&context=djls. Accessed 18 Oct 2022

[CR17] Chalifour NJ, Earle J, Macintyre L (2021) Coming of age in a warming world: The Charter's Section 15 (1) equality guarantee and youth-led climate litigation. Journal of Law & Equality 17:1-104. https://heinonline.org/HOL/PrintRequest?collection=journals&handle=hein.journals/jleq17&div=2&print=section&format=PDFsearchable&submit=Print%2FDownload&id=1. Accessed 17 Oct 2022

[CR18] Chenoweth E (2021) Civil resistance: What everyone needs to know. Oxford University Press, Oxford

[CR19] Chernaik* v. Brown* (2020) S066564. http://climatecasechart.com/climate-change-litigation/wp-content/uploads/sites/16/case-documents/2020/20201022_docket-S066564-_opinion.pdf. Accessed 17 Oct 2022

[CR20] Choquette C, Klaudt D, Lynes LS (2021) Climate change litigation in Canada. In: Sindico F and Mbengue MM (eds) Comparative climate change litigation: Beyond the usual suspects, Springer, pp. 153-197. https://link.springer.com/content/pdf/10.1007%2F978-3-030-46882-8.pdf. Accessed 2 Mar 2022

[CR21] Chung E (2021) There’s a good chance we’ll miss our climate goals. So what’s the point in setting them? CBC News. https://www.cbc.ca/news/science/climate-change-targets-faq-1.6237074. Accessed 14 Mar 2022

[CR22] Climate Change Litigation Databases (2018) *Future Generations v. Ministry of the Environment and Others [DemandaGeneraciones Futuras v. Minambiente].* http://climatecasechart.com/climate-change-litigation/non-us-case/future-generation-v-ministry-environment-others/. Accessed 15 Mar 2022

[CR23] Climate Change Litigation Databases (2022a) Canada. http://climatecasechart.com/non-us-jurisdiction/canada/ Accessed 3 March 2022

[CR24] Climate Change Litigation Databases (2022b) The right to a healthy environment, Climate Change Litigation Databases. http://climatecasechart.com/non-us-case-category/right-to-a-healthy-environment/. Accessed 20 Nov 2022

[CR25] Climate Change Litigation Databases (2019) *Milieudefensie et al. v. Royal Dutch Shell plc.* http://climatecasechart.com/climate-change-litigation/non-us-case/milieudefensie-et-al-v-royal-dutch-shell-plc/. Accessed 4 Mar 2022

[CR26] Cohen M, Dagenais MO (2021) The implementation of economic, social and cultural rights in Canada: Between utopia and reality. Const Rev 7:26. 10.31078/consrev7l2. Accessed 15 Oct 2022

[CR27] Council of Europe Portal (nd) The evolution of human rights. https://www.coe.int/en/web/compass/the-evolution-of-human-rights. Accessed 15 Oct 2022

[CR28] Dale A, Robinson J, King L, Burch S, Newell R, Shaw A, Jost F (2020) Meeting the climate change challenge: Local government climate action in British Columbia, Canada. Climate Policy 20(7):866-880. 10.1080/14693062.2019.1651244. Accessed 15 Mar 2022

[CR29] Daly A (2022) Climate competence: Youth climate activism and its impact on international human rights law. SSRN. https://papers.ssrn.com/sol3/papers.cfm?abstract_id=4018470. Accessed 20 Nov 2022

[CR30] *Daniel Billy and others v Australia* [Torres Strait Islanders Petition] (2019) CCPR/C/135/D/3624/2019. http://climatecasechart.com/non-us-case/petition-of-torres-strait-islanders-to-the-united-nations-human-rights-committee-alleging-violations-stemming-from-australias-inaction-on-climate-change/. Accessed 15 Mar 2022

[CR31] Davies G (2020) Climate change and reversed intergenerational equity: The problem of costs now, for benefits later. Climate Law 10(3-4):266-281. https://brill.com/view/journals/clla/10/3-4/article-p266_266.xml. Accessed 16 Oct 2022

[CR32] De Schutter O (2012) Special Rapporteur on the Right to Food, Mission to Canada, 6 to 16 May 2012. https://foodsecurecanada.org/sites/foodsecurecanada.org/files/20120321_SRRTF_Aide-m%C3%A9moire_Canada.pdf. Accessed 15 Feb 2022

[CR33] Desai D (2009) Courting legitimacy: Democratic agency and the justiciability of economic and social rights. Interdisciplinary Journal of Human Rights Law 4:25-42. https://heinonline.org/HOL/PrintRequest?collection=journals&handle=hein.journals/ijhrl4&div=5&print=section&format=PDFsearchable&submit=Print%2FDownload&id=27. Accessed 3 Feb 2022

[CR34] Elson D (2012) The reduction of the UK budget deficit: A human rights perspective. International Review of Applied Economics 26(2):177-190. 10.1080/02692171.2011.640315. Accessed 15 Mar 2022

[CR35] Environment and Climate Change Canada (2019) Canada’s Changing Climate Report. https://changingclimate.ca/site/assets/uploads/sites/2/2020/06/CCCR_FULLREPORT-EN-FINAL.pdf. Accessed 16 Mar 2022

[CR36] Environment and Climate Change Canada (2021) Canadian Environmental Sustainability Indicators: Progress towards Canada’s greenhouse gas emissions reduction target. https://www.canada.ca/en/environment-climate-change/services/environmental-indicators/progress-towards-canada-greenhouse-gas-emissions-reduction-target.html. Accessed 17 Mar 2022

[CR37] *Environnement Jeunesse c. Procureur général du Canada* (2019) QCCS 2885 (CanLII). https://canlii.ca/t/j1ghh. Accessed 17 Mar 2022

[CR38] Erratum to: Recent youth-led and rights-based climate change litigation in Canada: Reconciling justiciability, charter claims and procedural choices (2021) Journal of Environmental Law (20211207). 10.1093/jel/eqab036. Accessed 16 Mar 2022

[CR39] Feasby C, de Vlieger D, Huys M (2020) Climate change and the right to a healthy environment in the Canadian Constitution. Alberta Law Review 58(2):213-248. https://heinonline.org/HOL/PrintRequest?collection=journals&handle=hein.journals/alblr58&div=12&print=section&format=PDFsearchable&submit=Print%2FDownload&id=213. Accessed 21 Mar 2022

[CR40] Finn C (2002) The justiciability of administrative decisions: A redundant concept? Federal Law Review 30(2):239-263. 10.3316/agispt.20024517. Accessed 6 Mar 2022

[CR41] Fredman S (2005) Providing equality: Substantive equality and the positive duty to provide. South African Journal on Human Rights 21(2):163-190. 10.1080/19962126.2005.11865132. Accessed 14 Mar 2022

[CR42] Fredman S (2006) From deference to democracy: The role of equality under the Human Rights Act 1998. Oxford Legal Studies Research Paper No. 5/2006, Law Quarterly Review 122:53-81. https://ssrn.com/abstract=894252. Accessed 14 Mar 2022

[CR43] *Friends of the Earth v. Canada (Environment)* (2009) FCA 297 (CanLII). https://canlii.ca/t/26970. Accessed 15 Oct 2022

[CR44] Galanter M (1983) The radiating effects of courts. Empirical Theories of Courts 117-42. https://uwlaw-omeka.s3.us-east-2.amazonaws.com/original/9455adc48c549c053d5c80109861843932431e4a.pdf. Accessed 16 Oct 2022

[CR45] Gary MZ, Wilkie C, Baker D (2010) The law as it affects people with disabilities: A case study paper on rights to supports prepared for the Law Commission of Ontario: Final Paper. Bakerlaw. https://www.lco-cdo.org/wp-content/uploads/2010/11/disabilities_bakerlaw.pdfd. Accessed 4 Feb 2022

[CR46] Gentili G (2013) Protecting rights in a ‘worldwide rights culture’: An empirical study of the use of foreign precedents by the Supreme Court of Canada (1982–2010). In: Groppi T. and Ponthoreau MC (eds) The use of foreign precedents by constitutional judges. Oxford University Press, Oxford, pp 39-68. https://ssrn.com/abstract=2246364. Accessed 14 Mar 2022

[CR47] Gold K (2019) They’ve taken baby steps: Professor slams Ottawa’s housing strategy. The Globe and Mail. https://www.theglobeandmail.com/real-estate/vancouver/article-theyve-taken-baby-steps-housing-professor-slams-liberals-approach/. Accessed 15 Mar 2022

[CR48] Gönenç D (2019) Litigation process of social movements as a driver of norm transformation? Cambridge Review of International Affairs 32(1):43-60. 10.1080/09557571.2018.1556245. Accessed 16 Mar 2022

[CR49] *Gosselin v Quebec (Attorney General) *(2002) Supreme Court of Canada 84*.* https://scc-csc.lexum.com/scc-csc/scc-csc/en/item/2027/index.do. Accessed 15 Feb 2022

[CR50] Government of Canada (2021) Prime Minister Trudeau announces increased climate ambition. https://pm.gc.ca/en/news/news-releases/2021/04/22/prime-minister-trudeau-announces-increased-climate-ambition. Accessed 4 Feb 2022

[CR51] Gowling WLG (2020) *Gosselin v Quebec (Attorney General) *(2002 Supreme Court of Canada 84). https://scc-csc.lexum.com/scc-csc/scc-csc/en/item/2027/index.do. Accessed 14 Mar 2022

[CR52] Han H, Ahn SW (2020) Youth mobilization to stop global climate change: Narratives and impact. Sustainability 12(10):4127. 10.3390/su12104127. Accessed 15 Feb 2022

[CR53] Harvey D (1996) Justice, nature and the geography of difference. Oxford, Blackwell.

[CR54] Heffernan T, Faraday F, Rosenthal P (2015) Fighting for the right to housing in Canada. Journal of Law & Social Policy 24:10-45. https://digitalcommons.osgoode.yorku.ca/jlsp/vol24/iss1/2/. Accessed 15 Mar 2022

[CR55] Hilson C (2002) New social movements: The role of legal opportunity. Journal of European Public Policy 9(2):238-255. 10.1080/13501760110120246. Accessed 17 Mar 2022

[CR56] Hilson C (2010) Climate change litigation: A social movement perspective. *SSRN.* https://papers.ssrn.com/sol3/papers.cfm?abstract_id=1680362. Accessed 17 Mar 2022

[CR57] Hilson C (2022) The role of narrative in environmental law: The nature of tales and tales of nature. Journal of Environmental Law 34(1):1-24. 10.1093/jel/eqab043. Accessed 20 Nov 2022

[CR58] Hulchanski D (2021) Housing for all? What is the objective of Canada’s housing system? Oral presentation, Factor-Inwentash Faculty of Social Work, University of Toronto, Toronto, Ontario, Canada. http://neighbourhoodchange.ca/documents/2021/11/housing-for-all-canada-housing-system.pdf. Accessed 20 Nov 2022

[CR59] Human Rights Council (HRC) (2022) Views adopted by the Committee under article 5 (4) of the Optional Protocol, concerning communication No. 3624/2019 [Advanced unedited version]. https://tbinternet.ohchr.org/Treaties/CCPR/Shared%20Documents/AUS/CCPR_C_135_D_3624_2019_34335_E.docx. Accessed 20 Nov 2022

[CR60] Ignatief M (2000) The rights revolution. CBC Massey Lectures, Anansi

[CR61] *Indigenous Communities of the Lhaka Honhat (Our Land) Association v. Argentina* (2020) The Inter-American Court of Human Rights. https://www.corteidh.or.cr/docs/casos/articulos/seriec_400_ing.pdf. Accessed 20 Nov 2022

[CR62] Indigenous Services Canada (2022) Agreements-in-Principle reached on compensation and long-term reform of First Nations child and family services and Jordan’s Principle. https://www.canada.ca/en/indigenous-services-canada/news/2022/01/agreements-in-principle-reached-on-compensation-and-long-term-reform-of-first-nations-child-and-family-services-and-jordans-principle.html. Accessed 20 Nov 2022

[CR63] Intergovernmental Panel on Climate Change (IPCC) (2022) Climate Change 2022: Impacts, Adaptation and Vulnerability. Summary for Policymakers. https://report.ipcc.ch/ar6wg2/pdf/IPCC_AR6_WGII_FinalDraft_FullReport.pdf. Accessed 20 Nov 2022

[CR64] iPolitics (2021) Net zero: Calgary latest city to declare climate emergency. https://ipolitics.ca/2021/11/17/net-zero-calgary-latest-city-to-declare-climate-emergency/. Accessed 15 Mar 2022

[CR65] Jacklin C, Eidse-Rempel K (2021) Case brief: *Canada (Attorney General) v. First Nations Child and Family Caring Society of Canada, *2021 FC 969. DGW. https://www.dgwlaw.ca/case-brief-canada-attorney-general-v-first-nations-child-and-family-caring-society-of-canada-2021-fc-969/. Accessed 20 Nov 2022

[CR66] Jackman M (2019) One step forward and two steps back: Poverty, the Charter and the legacy of Gosselin. National Journal of Constitutional Law 39(1):85-121. https://www.proquest.com/docview/2171578102?pq-origsite=gscholar&fromopenview=true. Accessed 5 Mar 2022

[CR67] Jenkins JC, Wallace M, Fullerton AS (2008) A social movement society?: A cross-national analysis of protest potential. International Journal of Sociology 38(3):12-35. 10.2753/IJS0020-7659380301. Accessed 14 Mar 2022

[CR68] Jodoin S, Faucher R, Lofts K (2018) Look before you jump: Assessing the potential influence of the human rights bandwagon on domestic climate policy. In: S Duyck, S Jodoin, A Johl (eds) Routledge handbook of human rights and climate governance, Routledge, London, Chapter 11

[CR69] Jost F, Dale A, Newell R, Robinson J (2020) Evaluating development path changes using a novel climate action assessment framework in three municipalities in British Columbia, Canada. Environmental Science & Policy 114:410-421. 10.1016/j.envsci.2020.09.007. Accessed 3 Mar 2022

[CR70] *Juliana v. United States* (2020) 947 F.3d 1159 (9th Cir)

[CR71] Kelley D (2008) A life of one’s own: Individual rights and the welfare state. In: H Steiner, P Alston, R Goodman (eds) International human rights in context. Oxford University Press, Oxford

[CR72] Kirkup A, Evans T (2009) The myth of Western opposition to economic, social, and cultural rights? A reply to Whelan and Donnelly. Human Rights Quarterly 31:221. https://www.jstor.org/stable/20486741. Accessed 20 Nov 2022

[CR73] Krommendijk J (2021) Beyond Urgenda: The role of the ECHR and judgments of the ECtHR in Dutch environmental and climate litigation. RECIEL 00:1-15. 10.1111/reel.12405. Accessed 15 Mar 2022

[CR74] *La Rose et al. v. Canada* (2021) A-289–20 (Memorandum of Fact and Law of the Appellants). Federal Court of Appeal. http://climatecasechart.com/climate-change-litigation/wp-content/uploads/sites/16/non-us-case-documents/2021/20210503_T-1750-19_appeal.pdf. Accessed 16 Mar 2022

[CR75] *La Rose v. Her Majesty the Queen* (2019) T-1750–19. http://climatecasechart.com/climate-change-litigation/non-us-case/la-rose-v-her-majesty-the-queen/. Accessed 17 Mar 2022

[CR76] *Lagos del Campo v. Peru *(2017) Case No. 12.795, Judgment of 31 August 2017. ESCR-Net. https://www.escr-net.org/caselaw/2018/lagos-del-campo-vs-peru-case-no-12795-judgment-31-august-2017-preliminary-objections#:~:text=The%20Court%20ordered%20the%20State,intangible%20injury%E2%80%9D%2C%20since%20Mr. Accessed 20 Nov 2022

[CR77] Langford M (2008) The justiciability of social rights: From practice to theory. Social Rights Jurisprudence: Emerging Trends in International and Comparative Law 3:43-45. https://www.jus.uio.no/ior/english/people/aca/malcolml/justiciability_of_social_rights_from_practice_to_theory.pdf. Accessed 16 Oct 2022

[CR78] Leichenko R, Silva JA (2014) Climate change and poverty: Vulnerability, impacts, and alleviation strategies. Wiley Interdisciplinary Reviews: Climate Change 5(4):539-556. 10.1002/wcc.287. Accessed 16 Mar 2022

[CR79] Lewis B (2021) The potential of international rights-based climate litigation to advance human rights law and climate justice. Griffith Journal of Law & Human Dignity 9(1):5-27. https://griffithlawjournal.org/index.php/gjlhd/article/view/1213/1077. Accessed 20 Nov 2022

[CR80] Lister R (2013) Power, not pity: Poverty and human rights. Ethics and Social Welfare 7(2):109-123. 10.1080/17496535.2013.779002. Accessed 3 Feb 2022

[CR81] Marjanac S (2020) The role of climate change litigation: Interview with Sophie Marjanac, Interviewed by Andy Symington. Human Rights Defender 29:26-28. https://heinonline.org/HOL/P?h=hein.journals/hurighdef29&i=115. Accessed 15 Mar 2022

[CR82] *Mathur v. Her Majesty the Queen in Right of Ontario* (2021) ONSC 1624

[CR83] Matthews KR (2020) Social movements and the (mis)use of research: Extinction Rebellion and the 3.5% rule. Interface: A Journal For and About Social Movements 12(1):591-615. https://www.interfacejournal.net/wp-content/uploads/2020/07/Interface-12-1-Matthews.pdf. Accessed 20 Nov 2022

[CR84] May JR (2007) Climate change, constitutional consignment, and the political question doctrine. Denver University Law Review 85:919-960. https://heinonline.org/HOL/Page?handle=hein.journals/denlr85&id=927&collection=journals&index= Accessed 15 Mar 2022

[CR85] May JR, Daly E (2020) The indivisibility of human dignity and sustainability. In: Atapattu, S, Gonzalez CG, Seck S (eds), The Cambridge handbook on environmental justice and sustainable development. Cambridge University Press, Cambridge, pp. 1-25

[CR86] McCarthyTetrault (2021) Class action litigation on drinking water advisories on First Nations. https://www.mccarthy.ca/en/class-action-litigation-drinking-water-advisories-first-nations. Accessed 20 Nov 2022

[CR87] McLeod C (2017) Canada is seen as a global human rights beacon—but not so much, at home. Maclean’s. https://www.macleans.ca/opinion/canada-is-a-global-human-rights-beacon-but-not-so-much-at-home/. Accessed 5 Mar 2022

[CR88] Mejía-Lemos D (2022) The right to a healthy environment and its justiciability before the Inter-American Court of Human Rights: A critical appraisal of the *Lhaka Honhat v Argentina* judgement. Review of European, Comparative & International Environmental Law 31(2):317-324. 10.1111/reel.12438. Accessed 15 Mar 2022

[CR89] Muchlinski P (2020) Corporate liability for breaches of fundamental human rights in Canadian law: Nevsun Resources Limited v Araya. Series 2 Vol 1 Amicus Curiae 505. https://heinonline.org/HOL/LandingPage?handle=hein.journals/amcrae2019&div=53&id=&page=. Accessed 4 Mar 2022

[CR90] Murdoch D (2002) Welfare rights in a modern state: A theoretical approach to Gosselin v. Quebec. Journal of Law & Equality 1:25. https://heinonline.org/HOL/P?h=hein.journals/jleq1&i=25. Accessed 15 Mar 2022

[CR91] Mutua M (2016) Human rights standards: Hegemony, law, and politics. State University of New York Press, New York

[CR92] *National Housing Strategy Act. S.C.* (2019) c. 29, s. 313. https://laws-lois.justice.gc.ca/eng/acts/n-11.2/FullText.html. Accessed 15 Mar 2022

[CR93] *Neubauer et al. v Germany *(2021) Case No. BvR 2656/18/1, BvR 78/20/1, BvR 96/20/1, BvR 288/20 - Germany. https://www.acrisl.org/casenotes/m2ll8m8skjpglk8-83mk2-k5yza-dcafy-x5ztr-bjfxk-c9y55-5ryfp. Accessed 20 Nov 2022

[CR94] *Nevsun Resources Ltd. v. Araya* (2020) SCC 5, Canadian Legal Information Institute. https://canlii.ca/t/j5k5j. Accessed 15 Mar 2022

[CR95] Nicholson S, Chong D (2011) Jumping on the human rights bandwagon: How rights-based linkages can refocus climate politics. Global Environmental Politics 11(3):121-136. 10.1162/GLEP_a_00072. Accessed 15 Mar 2022

[CR96] Noonan D (2018) Imagining different futures through the courts: A social movement assessment of existing and potential new approaches to climate change litigation in Australia. University of Tasmania Law Review 37(2):25-69. 10.3316/informit.000683752372871. Accessed 4 Feb 2022

[CR97] OHRC (1966a) International Covenant on Economic, Social and Cultural Rights. https://www.ohchr.org/en/instruments-mechanisms/instruments/international-covenant-economic-social-and-cultural-rights. Accessed 3 Feb 2022

[CR98] OHRC (1966b) International Covenant on Political and Civil Rights. https://www.ohchr.org/en/instruments-mechanisms/instruments/international-covenant-civil-and-political-rights. Accessed 3 Feb 2022

[CR99] OHRC (1993) Vienna Declaration and Programme of Action. https://www.ohchr.org/en/resources/educators/human-rights-education-training/18-vienna-declaration-and-programme-action-1993. Accessed 3 Feb 2022

[CR100] Orangias J (2021) Towards global public trust doctrines: An analysis of the transnationalisation of state stewardship duties. Transnational Legal Theory 12(4):550-586. 10.1080/20414005.2021.2006030. Accessed 17 Mar 2022

[CR101] Parker L, Mestre J, Jodoin S, Wewerinke-Singh M (2021) When the kids put climate change on trial: Youth-focused rights-based climate litigation around the world. Journal of Human Rights and Environment. https://ssrn.com/abstract=3960982. Accessed 14 Mar 2022

[CR102] Parliament of Canada (2019) Motion: Government Business No. 29 (National climate emergency). Vote No. 1366. 42nd Parliament, 1st Session. Sitting No. 435 - Monday, June 17, 2019. https://www.ourcommons.ca/Parliamentarians/en/votes/42/1/1366/. Accessed 15 Oct 2022

[CR103] Parliamentary Budget Officer (2021) Federal Program Spending on Housing Affordability in 2021. https://www.pbo-dpb.gc.ca/en/blog/news/RP-2122-014-S--federal-program-spending-housing-affordability-in-2021--depenses-federales-programmes-consacrees-abordabilite-logement-2021. Accessed 16 Oct 2022

[CR104] Peel J, Lin J (2019) Transnational climate litigation: The contribution of the Global South. American Journal of International Law 113(4):679-726. 10.1017/ajil.2019.48. Accessed 16 Oct 2022

[CR105] Peel J, Markey-Towler R (2022) Recipe for success? Lessons for strategic climate litigation from the *Sharma*, *Neubauer*, and *Shell* cases. German Law Journal 22(8):1484-1498. 10.1017/glj.2021.83. Accessed 20 Nov 2022

[CR106] Peel J, Osofsky HM (2018) A rights turn in climate change litigation? Transnational Environmental Law 7(1):37-67. 10.1017/S2047102517000292. Accessed 16 Oct 2022

[CR107] Peel J, Osofsky HM (2020) Climate change litigation. Annual Review of Law and Social Science 16:21-38. 10.1146/annurev-lawsocsci-022420-122936. Accessed 17 Mar 2022

[CR108] Pierson P (2002) Coping with permanent austerity: Welfare state restructuring in affluent democracies. Revue française de sociologie 43(2):369-406. https://www.jstor.org/stable/pdf/3322510.pdf. Accessed 15 Feb 2022

[CR109] Pieterse M (2004) Coming to terms with judicial enforcement of socio-economic rights. South African Journal on Human Rights 20(3):383-417. https://hdl.handle.net/10520/EJC53156. Accessed 15 Feb 2022

[CR110] Raphael D (2006) Social determinants of health: Present status, unanswered questions, and future directions. International Journal of Health Services 36(4):651-677. 10.2190/3MW4-1EK3-DGRQ-2CRF. Accessed 13 Feb 2022 10.2190/3MW4-1EK3-DGRQ-2CRF 17175840

[CR111] Real Leaders (2019) Greta Thunberg: “I don’t want your hope”. https://real-leaders.com/greta-thunberg-i-dont-want-your-hope/. Accessed 15 Feb 2022

[CR112] Riches G (2002) Food banks and food security: Welfare reform, human rights and social policy. Lessons from Canada? Social Policy & Administration 36(6):648-663. 10.1111/1467-9515.00309. Accessed 15 Feb 2022

[CR113] Rodríguez-Garavito C (2020) Human rights: The Global South’s route to climate litigation. AJIL Unbound 114:40-44. 10.1017/aju.2020.4. Accessed 15 Mar 2022

[CR114] Routledge P (2003) Convergence space: process geographies of grassroots globalization networks. Transactions of the Institute of British Geographers 28(3):333-349. 10.1111/1475-5661.00096. Accessed 14 Mar 2022

[CR115] Savaresi A, Auz J (2019) Climate change litigation and human rights: Pushing the boundaries. Climate Law 9(3):244-262. 10.1163/18786561-00903006. Accessed 17 Mar 2022

[CR116] Scott C, Macklem P (1992) Constitutional ropes of sand or justiciable guarantees? Social rights in a new South African Constitution. University of Pennsylvania Law Review 141. https://papers.ssrn.com/sol3/papers.cfm?abstract_id=1162216. Accessed 15 Mar 2022

[CR117] Sen A (2009) *The idea of justice*. Belknap Press of Harvard University Press

[CR118] Setzer J, Byrnes R (2020) Global trends in climate change litigation: 2020 snapshot. Grantham Research Institute for Climate Change and Environment, London School of Economics. https://www.lse.ac.uk/granthaminstitute/wp-content/uploads/2020/07/Global-trends-in-climate-change-litigation_2020-snapshot.pdf. Accessed 14 Mar 2022

[CR119] Setzer J, Vanhala LC (2019) Climate change litigation: A review of research on courts and litigants in climate governance. Wiley Interdisciplinary Reviews: Climate Change 10(3):e580, 1-19. 10.1002/wcc.580. Accessed 15 Feb 2022

[CR120] *Sharma by her litigation representative Sister Marie Brigid Arthur v Minister for the Environment* (2021) FCA 560

[CR121] Siegel R (2004) The jurisgenerative role of social movements in United States constitutional law. https://law.yale.edu/sites/default/files/documents/pdf/Faculty/Siegel_Jurisgenerative_Role_of_Social_Movements.pdf. Accessed 15 Feb 2022

[CR122] Smith J (2012) Connecting social movements and political moments: Bringing movement building tools from global justice to Occupy Wall Street activism. Interface: A Journal For and About Social Movements 4(2):369-382. http://www.interfacejournal.net/wordpress/wp-content/uploads/2012/11/Interface-4-2-Smith.pdf. Accessed 15 Mar 2022

[CR123] Smith-Carrier T (2021). The (charitable) pantry is bare: A critical discourse analysis of Christmas food hamper programs in Canada. Critical Policy Studies 15(1):90-106. 10.1080/19460171.2020.1722190. Accessed 21 Nov 2022

[CR124] Smith-Carrier T, On J (In Press) Ambitious for change? A critical appraisal of the Canadian Indicator Framework of the Sustainable Development Goals. Journal of Human Rights Practice.

[CR125] Smith-Carrier T, Ross K, Kirkham J, Decker Pierce B (2017) Food is a right…Nobody should be starving on our streets: Perceptions of food bank usage in a mid-sized city in Ontario, Canada. Journal of Human Rights Practice 9(1):29-49. 10.1093/jhuman/huw021. Accessed 21 Nov 2022

[CR126] Smith-Carrier T, Montgomery P, Mossey S, Shute T, Forchuk C, Rudnick A (2020) Erosion of social support for disabled people in Ontario: An appraisal of the Ontario Disability Support Program (ODSP) using a human rights framework. Canadian Journal of Disability Studies 9(1): 1-30. 10.15353/cjds.v9i1.594. Accessed 21 Nov 2022

[CR127] Stuart D, Gunderson R, Petersen B (2020) The climate crisis as a catalyst for emancipatory transformation: An examination of the possible. International Sociology 35(4):433-456. 10.1177/02685809209150. Accessed 16 Mar 2022

[CR128] *Tanudjaja v. Canada (Attorney General)* (2014) ONCA 852, C57714. https://www.ontariocourts.ca/decisions/2014/2014ONCA0852.htm. Accessed 15 Mar 2022

[CR129] *Tataskweyak Cree Nation et al. v. Canada (Attorney General) *(2021) MBQB 275

[CR130] Tramel S (2018) Convergence as political strategy: Social justice movements, natural resources and climate change. Third World Quarterly 39(7):1290-1307. 10.1080/01436597.2018.1460196. Accessed 14 Mar 2022

[CR131] *Tsleil-Waututh v. Canada (Attorney General)* (2018) FCA 153

[CR132] UN (1948) Universal Declaration of Human Rights. https://www.un.org/en/about-us/universal-declaration-of-human-rights Accessed 15 Mar 2022

[CR133] UN (1997) Masstricht Guidelines on Violations of Economic, Social and Cultural Rights, Maastricht

[CR134] UN Committee on Economic, Social and Cultural Rights (CESCR) (1990) General Comment No. 3: The Nature of States Parties’ Obligations (Art. 2, Para. 1, of the Covenant). E/1991/23. https://www.refworld.org/docid/4538838e10.html. Accessed 15 Mar 2022

[CR135] UN Environment Programme (1993) *Minors Oposa v. Factoran*. https://leap.unep.org/countries/ph/national-case-law/minors-oposa-v-factoran. Accessed 15 Mar 2022

[CR136] UN Human Rights Committee (2022, September). Views adopted by the Committee under article 5 (4) of the Optional Protocol, concerning communication No. 3624/2019. https://tbinternet.ohchr.org/Treaties/CCPR/Shared%20Documents/AUS/CCPR_C_135_D_3624_2019_34335_E.docx. Accessed 20 Nov 2022

[CR137] UN News (2022) UN General Assembly declares access to clean and healthy environment a universal human right. https://news.un.org/en/story/2022/07/1123482 Accessed 20 Nov 2022

[CR138] *Urgenda Foundation v. The Netherlands* (2015) HAZA C/09/00456689 (24 June 2015); aff’d (9 October 2018) (District Court of the Hague, and The Hague Court of Appeal (on appeal)

[CR139] Whelan DJ (2010) Indivisible human rights: A history. University of Pennsylvania Press, Pennsylvania

[CR140] World Health Organization (2021) COP26 Special Report on Climate Change and Health: The Health Argument for Climate Change. https://www.who.int/publications/i/item/cop26-special-report. Accessed 15 Mar 2022

[CR141] WMO (2022) United in science: We are heading in the wrong direction. https://public.wmo.int/en/media/press-release/united-science-we-are-heading-wrong-direction. Accessed 20 Nov 2022

[CR142] Wright EO (2010) Envisioning real utopias. Verso, London

